# Chronic 17β-estradiol treatment improves negative valence, anhedonic profile, and social interactions in ovariectomized, middle-aged female rats

**DOI:** 10.3389/fnbeh.2025.1553501

**Published:** 2025-07-02

**Authors:** Cheryl D. Conrad, Dylan N. Peay, Sara Sladkova, Jinah L. Kim, Megan E. Donnay, Amanda M. Acuña, Kennedy E. Whittaker

**Affiliations:** Department of Psychology, Arizona State University, Tempe, AZ, United States

**Keywords:** estrogen improves negative valence, anhedonia and sociability in middle-age depression, anxiety, estrogen, sociability, Forced Swim Test (FST), sucrose preference

## Abstract

Women experience depression at nearly 2-fold higher rates than men, with middle-age during the menopausal transition being particularly vulnerable. Preclinical studies commonly focus on young adult or aged subjects and/or rely upon a few behavioral tasks. Given the highly variable and heterogenous nature of depression, the current study implemented a behavioral battery to assess whether estradiol (E2, endogenously expressed in women and rats) would improve depressive measures using the Research Domain Criteria (RDoC) for negative valence, anhedonia, sociability, and anxiety in early middle-aged, ovariectomized (OVX) female rats. F344-cdf rats were OVX and injected daily with E2 (3 μg/ml, or oil). Behavioral testing began after 14 days of injections, which continued throughout the study. E2 improved the depressive profile when using a composite metric for negative valence (immobility on the forced swim task, FST), anhedonia (duration to initiate grooming following sucrose splash and latency to initiate grooming with sucrose), sociability (time interacting toward a novel conspecific), and novelty-induced anxiety (time spent investigating marbles). Interestingly, FST immobility significantly and positively correlated with sucrose preference to show they were opposingly related: higher immobility on FST corresponded to more sucrose ingested. Also, time spent in a chamber with a novel conspecific was less informative than time directed at the conspecific. Other tasks, such as the marble bury test showed some hoarding behavior. These nuances revealed difficulties in assessing behaviors within and across studies, but overall showed that E2 improved the depressive-like syndrome (DLS) in middle-aged females based upon the RDoC.

## Introduction

Depression is twice as common in women than men (Kessler, 2003; Kessler et al., 2005; Seedat et al., 2009; Willi and Ehlert, 2019), with women's hormonal state being an important influence (Altshuler et al., 2001; Young and Korszun, 2010; Altemus et al., 2014; Albert, 2015). Women are also more likely to co-express anxiety disorders with depression (Altemus et al., 2014; Kalin, 2020), whereas men commonly co-express with substance abuse (Kornstein et al., 1995; Sagud et al., 2002; Marcus et al., 2005; Albert and Newhouse, 2019). The menopausal transition is a particularly vulnerable period in middle-aged women (Schmidt and Rubinow, 2009; Soares, 2017). During perimenopause, levels of ovarian hormones (estrogen and progesterone) fluctuate erratically and eventually settle at lower concentrations from reproductive maturity (Burger et al., 2007). Perimenopause coincides with hot flashes and night sweats, which overlap with increased vulnerability to depression and anxiety (Schmidt and Rubinow, 2009; Gordon et al., 2016; Soares, 2017; Gordon et al., 2019). Even women without a history of depression are at increased risk for developing a mood disorder during the middle-age transition (Cohen et al., 2006; Epperson et al., 2013) with hormone treatments helping to reduce depressive symptomology (Dwyer et al., 2020; Zhang J. et al., 2023). Consequently, middle-aged women undergoing menopause are at a particularly high risk for developing depression.

Animal models can help researchers better understand depression (Willner et al., 1992; Cryan et al., 2002; Willner and Mitchell, 2002), with growing support that the complex symptomology of depression entails extrapolating from a variety of models to understand depression's biology (Söderlund and Lindskog, 2018; Gururajan et al., 2019). However, the terminology used in preclinical research often differs from that used in clinical settings (Söderlund and Lindskog, 2018; Gururajan et al., 2019), prompting the National Institute of Mental Health to create the Research Domain Criteria (RDoC, Sanislow et al., 2010; Anderzhanova et al., 2017; von Mücke-Heim et al., 2023) to guide mental health investigations (National Institute of Mental Health, 2019). With regard to the RDoC negative valence systems involving aversive situations and positive valence systems involving reward, chronic stress is disruptive in male rodents, as measured by the forced swim task (FST), elevated plus maze (EPM), open field (OF), reward salience, and sucrose preference (SP, D'Aquila et al., 1994; Willner, 1997; Gregus et al., 2005; Brenes-Sáenz et al., 2006; Bondi et al., 2008; Huynh et al., 2011; Kukel'ova et al., 2018; Liu et al., 2018; Du Preez et al., 2020; Seewoo et al., 2020; Mao et al., 2022; Reddy et al., 2024). Interestingly, similar assessments in chronically stressed female rodents are inconsistent (Huynh et al., 2011; Hodes and Epperson, 2019), perhaps in part because studies using females are fewer than those using males (Czeh et al., 2016; Du Preez et al., 2021; Brandwein et al., 2023). This is especially true concerning the targeted demographic of middle-aged rodent models (Nakanishi et al., 2025). We recently reported that middle-aged OVX female rats exposed to daily stress levels of corticosterone, the endogenous stress steroid in rodents, co-expressed depressive- symptomology and poor working memory (Conrad et al., 2024). This conflicts with our prior studies showing chronic stress fails to disrupt cognition or depressive symptomology in young adult female rats (Conrad et al., 2003; McLaughlin et al., 2010; Huynh et al., 2011). Altogether, these findings support the idea that middle-age may be unique compared to young adulthood. Due to the higher clinical rates of depression and anxiety in women and aging individuals compared to men and young adults (Wu and Anthony, 2000; Kessler et al., 2007; Seedat et al., 2009; Schaakxs et al., 2018), more studies with females of different ages are needed, particularly at middle-age.

Ovarian hormones and aging can impact the systems related to the RDoC for social processes and negative valence in females. Compared to sham or estrogen-treated females, ovariectomized (OVX) female rats show a depression-like syndrome (DLS, von Mücke-Heim et al., 2023), characterized by social withdrawal (Hlinak, 1993; Markham and Juraska, 2007), immobility in the FST (Hiroi et al., 2016), and heightened anxiety in the OF and EPM (Frye and Walf, 2004; McLaughlin et al., 2008; Conrad et al., 2012; Hiroi et al., 2016). Furthermore, the effects of OVX and estrogens on negative valence systems, positive valence systems, and cognitive systems depend upon many factors, such as the age of the subject when OVX was performed and timing from OVX to behavioral testing (Markham and Juraska, 2007; McLaughlin et al., 2008; Benmansour et al., 2016; Grigoryan, 2022). Aging female rodents, in particular, express high levels of negatively valenced behaviors (Boyer et al., 2019; Nolte et al., 2019). Consequently, OVX and aging in female rodents may be useful for modeling depression for particularly vulnerable demographics.

These age-related hormonal changes in females induce an endophenotype that may underlie the RDoC of negative valence-related behavioral changes. This is characterized by increased neuroinflammation, hypothalamic-pituitary-adrenal (HPA) axis dysregulation, and altered activity in brain regions involved in emotional regulation. The loss of ovarian hormones in aged mice increases neuroinflammation (Benedusi et al., 2012) and extends beyond depression to other psychiatric disorders, HPA axis dysfunction, and emotional dysregulation as well (Benedusi et al., 2012; Rhie et al., 2020; Troubat et al., 2021). Moreover, endogenous estrogen regulates the HPA axis to help diminish inflammatory mediator expression (Liu et al., 2012; Villa et al., 2016). Finally, the loss of endogenous estrogen with aging alters brain activity in structures integral to emotion processing and regulation, such as the dorsolateral prefrontal cortex and amygdala, as demonstrated by functional neuroimaging findings comparing pre- peri- and post-menopausal women (Albert et al., 2021). Consequently, targeting estrogens in middle-age represents a critical endophenotype that is important to understand in the treatment of depression.

Preclinical studies show that estrogens exert some beneficial influences on negative valence and cognition. Negative valence behaviors are reduced with estrogens in OVX female rodents (Hlinak, 1993; Spiteri and Agmo, 2009; Garcia et al., 2017) and following the birth of pups (Galea et al., 2001). For cognition, 17b-estradiol (E2), provides some benefits for reference, short-term, and working memory (Bimonte and Denenberg, 1999; McLaughlin et al., 2008; Luine, 2014; Frick et al., 2018; Koebele et al., 2020; Bowman et al., 2022). However, most studies focus on young or aged adults, with the middle-aged demographic being understudied, despite the heightened risk for developing depression during this time period in women. Moreover, those studies that examined middle-aged OVX female rats were limited to assessing a few behaviors in response to estrogens, but nevertheless show that E2 improved performance on the FST (Benmansour et al., 2016), enhanced cognition on spatial tasks (Bimonte-Nelson et al., 2006; Talboom et al., 2008; Koebele et al., 2020) and reduced thigmotaxia on the OF (Renczes et al., 2020). To investigate E2 influences on affective behavior, which is multifaceted with highly variable positive and negative valence systems (Slattery and Cryan, 2014; Gururajan et al., 2019; National Institute of Mental Health, 2019), the current study included a variety of measures to assess affective behavioral changes during the particularly sensitive period of early stages of middle-age in female rodents. Negative valence for acute threat and potential threat was selected because depression distorts the processing of negatively valenced emotional stimuli (Fossati, 2018). Positive valence for reward was selected because anhedonia is one of the key symptoms of depression (DSM-5). Social processes for affiliation were selected because social functioning is intertwined with depression and another significant aspect of this disorder (Kupferberg et al., 2016; Steare et al., 2025; Thompson et al., 2025). We thus tested the hypothesis that E2 treatments would improve the negative valence, positive valence, and social outcomes in our rodent model of OVX, middle-aged female rats.

## Methods

### Subjects

Arizona State University Institutional Animal Care and Use Committee approved the procedures, which align with the Guide for the Care and Use of Laboratory Animals. Thirty-four middle-aged (9–10 months) virgin female Fischer-344-cdf rats (National Institute on Aging, Hollister, CA, USA) were pair-housed in standard laboratory cages (21–22°C) with Sani-Chip bedding (Tekland, #7090, Inotiv, Lafayette, IN). Except where noted below, rats were allowed food and reverse osmosis (RO) water *ad libitum*. Rats were housed on a reverse 12:12 light cycle; lights off at 07:00. All procedures occurred during the dark phase of the light cycle. Fischer-344 cdf inbred rats were selected due to the National Institute on Aging's recommendation to use the strain for aging research due to their longevity and well-defined aging patterns (Koebele and Bimonte-Nelson, 2016). In addition, the age of the rats was chosen so that the rats would be behaviorally tested in the beginning of estropause, the equivalent of human perimenopause. In the rat, estropause occurs around 9 to 12 months of age (Koebele and Bimonte-Nelson, 2016).

### Ovariectomy surgery

All female rats were OVX after habituating to the animal colony room. Rats were prophylactically injected with meloxicam (1 mg/kg, i.p.) and buprenorphine (0.03 mg/kg, i.p.) for pain and anesthetized with isoflurane gas. The rats were laid prone on a heating pad, and eyes covered with ophthalmic ointment. The dorsal surgical sites were shaved and prepared with antiseptic surgical scrub, alternating with 70% ethanol three times prior to incision. For each side, an incision was made and gently opened dorsolaterally to 1.0 to 1.5 cm in the skin and peritoneum, caudal to the last rib. The ovary and uterine horn tip were ligated with dissolvable vicryl sutures (Stoelting Co.), the uterine horn cut with scissors, and then the ovary was gently extracted by grasping surrounding fat without touching the ovary. The muscle was closed with dissolvable sutures, marcaine applied, and the skin closed with wound clips (George Teimann & Co.). Post-surgical care included treating the surgical site with antibiotic ointment and monitoring the rats under a heat lamp until they were able to move and groom. Injections of meloxicam and buprenorphine continued daily for 2 days. Buprenorphine and its active metabolite, norbuprenorphine, undergo 4 to 5 half-lives within 24 h (Ohtani et al., 1994), indicating that they would be cleared by the start of the experimental treatments. Rats were single-housed for 3 to 5 days and then reunited with their prior cage-mates. Wound clips were removed 10–14 days after surgery. One rat expired due to surgical complications leaving 31 rats for experimental procedures (the remaining two rats were reserved for the social interaction part of the study). The same 31 rats were used for all tests.

### Estradiol (E2) treatment

E2 (Sigma Aldrich, 3 μg/ml in 0.1 ml sesame oil, s.c.) treatment began 21 days after OVX and continued daily throughout behavioral testing. The dose of E2 was chosen for several reasons. First, 3 μg leads to circulating levels of estradiol within the diestrous range in OVX female rats (Viau and Meaney, 1991). Second, this dose and treatment regimen successfully improves spatial working memory in middle-aged OVX female rats (Koebele and Bimonte-Nelson, 2015; Prakapenka et al., 2018). Third, females in the proestrous phase of the estrous cycle show elevated locomotor activity (Krentzel et al., 2020), which can confound interpretation when behaviors require locomotion. However, the diestrous-ranged dose of 3 μg dose has previously failed to alter locomotion on the OF task (Hiroi et al., 2016) and other studies using a slightly higher dose of 5 μg also failed to observe significant changes in locomotion (Fedotova, 2013, 2014). Thus, the diestrous-range dose of 3 μg was used in the current investigation to further examine its potential beneficial effects and to avoid confounds from locomotor effects. Rats were randomly assigned to receive daily E2 or sesame oil vehicle (VEH, Sigma-Aldrich) injections. VEH and E2 injections occurred between 7:00–8:00 am and behavioral tasks were conducted at least an hour after the final treatment injection.

### Timeline for treatments

#### Delay between OVX and E2 treatment

The 21-day delay between OVX and E2 treatment was chosen for several reasons. First, we wanted to ensure ovarian hormone clearance before E2 treatment began as estrogen clears within 1 to 2 weeks post-operatively and then E2 is typically low or undetectable in OVX rat plasma (Diaz Brinton, 2012). This also ensures that VEH-treated rats would be in a blank state to assess the effectiveness of E2 treatment without confounds from endogenous hormones (Prakapenka et al., 2018). Moreover, the decline in ovarian hormones is reflective of the pattern of estrogen depletion occurring during perimenopause and menopause (Burger et al., 2007). While a delay between OVX and E2 treatment can change the brain's responsiveness to E2 (Savonenko and Markowska, 2003; Daniel et al., 2006; Sherwin, 2007), our prior work found that initiating E2 treatment 3 weeks after OVX still provided sufficient time for E2 to influence the brain and behavior (McLaughlin et al., 2008). Finally, others reported that OVX rats show a progressively higher occurrence of anxiety-like and depressive-like behaviors at 3 weeks vs. 1 week post-OVX (Rodriguez-Landa, 2022).

#### Delay between E2 treatment and behavioral testing

E2 treatments started 1 week before rats began acclimation to the two choice drinking bottles. The first behavioral assessment for SP occurred when E2 treatments were already underway for 2 weeks to reduce potential, acute effects of E2 on behavior. By the time the rats were tested on the SP test, those receiving E2 injections would have already developed a steady level of E2 (Stanczyk et al., 2013).

#### Task order

**Figure 1:**
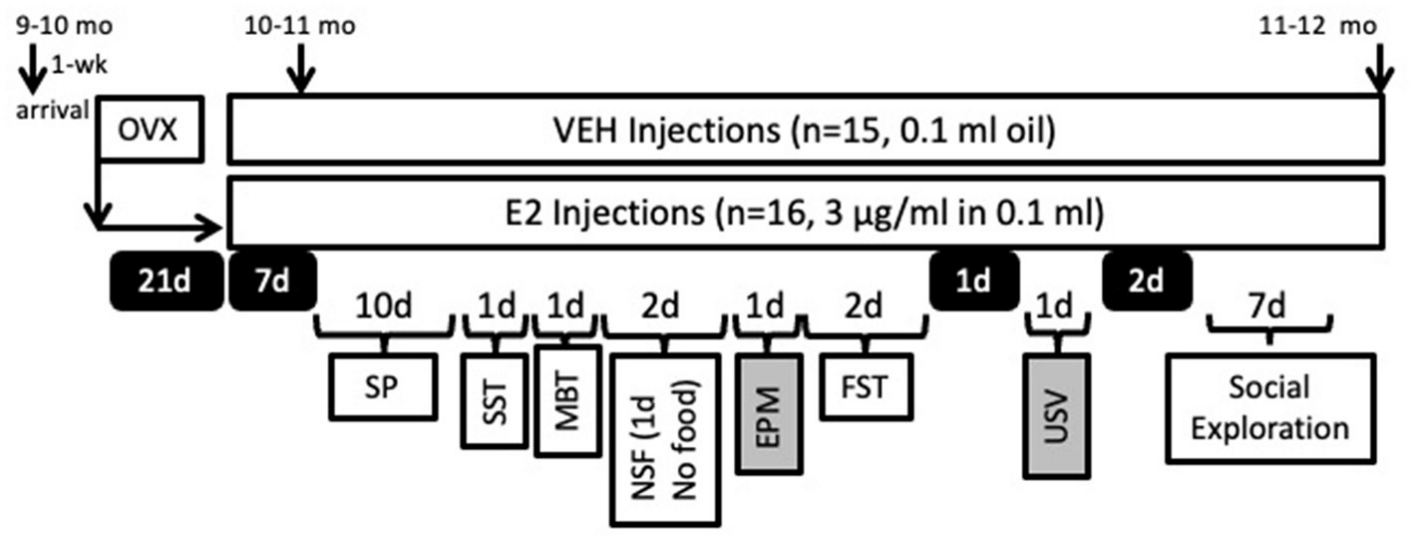
Timeline of the experimental manipulations relative to the behavioral testing schedule. Female rats were ovariectomized (OVX) and then given 21 days to recover without experimental manipulations. Rats were randomly assigned to estradiol (E2, 3 μg/ml, s.c.) or sesame oil vehicle treatments (VEH, s.c.) and were 10–11 months of age at the start of the hormone treatment. Rats were tested in the behavioral tasks in the order listed. Days off from behavioral testing are denoted in black boxes with white lettering and indicate the number of days without behavioral testing. Due to unforeseen circumstances, the elevated plus maze (EPM) and ultrasonic vocalization (USV) data were unusable and denoted with gray boxes. SP, Sucrose Preference; SST, Sucrose Splash Test; MBT, Marble Bury Task; NSF, Novelty Suppressed Feeding; FST, Forced Swim Test.

The task order was as follows: SP, sucrose splash test (SST), defensive marble bury task (MBT), novelty suppressed feeding (NSF), FST, and social exploration (Figure 1). The order was chosen based upon task availability and with some considerations. For the SP, the rats were acclimated for 7 days, which allowed them to be exposed for 2 weeks of E2 treatment and allowed blood levels of estrogen to stabilize before assessments were made (Stanczyk et al., 2013). The next tests were kept in the same order across rats with relatively increasing levels of task aversiveness (i.e., SP, MBT, NSF, EPM, FST), as our past work found that more aversive measures impacted behavior when behavioral testing occurred back to back (Peay et al., 2020). The FST was therefore performed toward the end of all testing due to its inherent aversiveness. Finally, social exploration was performed after a few days from the FST when the tasks became available.

### General testing procedures

Each procedure was performed in a room that was novel to the rats. There were four rooms and sections of a room were partitioned with curtains and changed with unique cues to provide a novel environment for each test. To reduce potential behavioral effects that could result from rats being exposed to various experimenters, unwashed t-shirts were placed in the testing room, concealed from the rat's view during testing (Sorge et al., 2014). To provide white noise and ensure the dispersion of odors, fans were placed in testing rooms. For the defensive MBT, FST, and social exploration, behaviors were recorded with GoPro cameras (Hero3) for offline scoring later by investigators who were blinded to the experimental conditions. Cameras were installed overhead and/or to the side. Testing order was counterbalanced across groups throughout the day.

For behavioral tests outside the colony room (defensive MBT, NSF, FST, and social exploration), rats were transported with their cage-mates in their home cages using a cart and acclimated in a quiet room (red lights) located nearby to the testing rooms and given at least 30 min before they were tested. Due to unforeseen issues, data from the EPM and ultrasonic recording were unusable. The RDoC domains and constructs were organized based upon the described characteristics (Söderlund and Lindskog, 2018). The first set of constructs are found in depression symptomology and include negative valence construct (acute threat) positive valence construct (anhedonia), and social processes construct (disrupted affiliation). Additionally, the last negative valence construct (potential or perceived harm, assessed as anxiety) is highly comorbidly expressed in women with depression (Altemus et al., 2014; Kalin, 2020).

### RDoC domain: negative valence behaviors (construct = acute threat)

#### Forced Swim Test

The FST was used because of past work that described it as reflecting behavioral despair (Porsolt et al., 1977; Lucki, 1997; Lino-de-Oliveira et al., 2005; Slattery and Cryan, 2012; Yankelevitch-Yahav et al., 2015), although other interpretations include altered coping strategies (Nestler and Hyman, 2010; Molendijk and de Kloet, 2015; Armario, 2021; Gencturk and Unal, 2024) and psychomotor retardation (Unal and Canbeyli, 2019). These differences in interpretation necessitate multiple assessments of depressive-like behaviors within the same subjects, as done in the present work. Importantly, antidepressants reliably improve FST performance, giving it predictive, if not construct validity (Brandwein et al., 2023) and why preclinical research still continues to include it in their behavioral batteries (Huang et al., 2023; Montazeri et al., 2023; Farinha-Ferreira et al., 2024; Liang et al., 2024; Reddy et al., 2024; Zubkov et al., 2024; Lei et al., 2025; Valente et al., 2025). However, caution is recommended to avoid overinterpretation of the results when they stand alone (Willner and Mitchell, 2002) and the reason why the current study used multiple behavioral measures to create a DLS.

The FST protocol consisted of 2 days of exposure with 10 min of acclimation on day 1 without recording and 5 min of testing on day 2 with recording, which is consistent with FST procedures for rats (Brandwein et al., 2023; Gencturk and Unal, 2024) and our past work (Conrad et al., 2024). The apparati consisted of four clear cylindrical Plexiglas tanks (45 cm height, 20 cm diameter) filled with water (23°C) to about 30 cm, such that when floating, a rat could not touch the bottom of the tank. Partitions were used to obscure rats from viewing each other and the experimenters, while allowing four rats to be tested simultaneously.

On the 1^st^ day, each rat was placed into individual tanks for 10 min and then immediately removed and placed under a heat lamp for 1-h prior to being returned to the housing colony. The FST tanks were rinsed and refilled with fresh water for each rat. On day 2 of testing, each rat was placed back into the tank for 5 min and behavior was recorded. After testing, each rat was again placed under a heat lamp prior to being returned to the housing colony.

FST behaviors quantified included time spent swimming, climbing, and being immobile. Swimming was defined as paw movement underwater as well as diving behavior. Climbing was defined as actively pawing at the walls of the apparatus with paws breaching the water surface. Immobility was defined as floating or the lack of movement. Rats that spend less time swimming and climbing, and more time immobile are considered to have elevated depressive-like profiles (Detke et al., 1995; Lino-de-Oliveira et al., 2005; Huynh et al., 2011; Slattery and Cryan, 2012).

### RDoC domain: positive valence behaviors (construct = anhedonia)

#### Sucrose preference

SP was utilized to measure hedonic profile by exposing the rats to a free choice between a highly palatable sucrose solution or their standard drinking water. A reduced preference for the sucrose solution served as an indicator of anhedonia (Katz, 1982; Willner, 2005; Liu et al., 2018; Reddy et al., 2024). This study utilized an extended SP assessment (10 days) without the need for food or water deprivation procedures (Taliaz et al., 2010; Najjar et al., 2018), consistent with our past work (Conrad et al., 2024).

Rats were single housed and habituated to two drinking bottles: 2% sucrose in RO water in one bottle and RO water in another for 3 days while food was provided *ad libitum*. After 3 days, the sucrose concentration was reduced to 1% sucrose and rats continued to have access to two bottles: 1% sucrose and RO for 7 days. The bottle locations were counterbalanced and swapped daily to account for potential side bias. The amount of liquid consumed was measured daily and solutions were replaced every 2 to 3 days. The amount of sucrose consumed was measured over the last 3 days of the 1% sucrose test.


$$\begin{array}{l} \text{An SP index }=\text{\% }\left(\text{volume of sucrose drink consumed}\right)\\ \;÷\;\left(\text{total volume of drink consumed}\right) \end{array}$$

The SP Index was assessed over the final 3 days of 1% sucrose testing and for the initial 3 days of 2% sucrose acclimation.

#### Sucrose splash test

The SST may reflect anhedonia through a reduction in reward-seeking behaviors (Bevins and Besheer, 2005; Willner, 2005), combined with a reduction in self-care. We implemented a version using 10% sucrose solution, based off of previous literature using rats and mice (Isingrini et al., 2010; Frisbee et al., 2015; Hu et al., 2017) and found to be effective in females (Hodes et al., 2015).

Testing occurred within an empty housing unit in the rat's regular colony room. A 10% sucrose solution (40 g sucrose in 400 ml RO water) was prepared a day before testing and placed in 200 ml spray bottles. Testing cages were prepared for each rat and consisted of a typical rat housing cage and rodent bedding. During testing, rats were transferred to the test cage and immediately sprayed twice: on the back of its head and the dorsal part of its shoulders/upper back. The housing unit doors were closed, and the rat was recorded for 5 min, before being returned to its home cage. Cagemates were tested simultaneously with the help of two investigators. Behaviors assessed included latency to begin grooming, total duration spent grooming, and number of grooming bouts.

### RDoC domain: systems for social processes (construct = disrupted affiliation)

#### Social exploration

The social interaction test was originally developed as a test of anxiety that was ethologically relevant in social animals, such as rats (File and Hyde, 1978; File and Seth, 2003). Exaggerated expression of stress-related fear behaviors in rats, such as freezing posture or a lack of exploration are likely representative of an anxious endophenotype pertinent to pathological anxiety (Bakshi and Kalin, 2002). Moreover, social withdrawal is an element of depression (American Psychiatric Association, 2013), suggesting that the construct of sociability overlaps with features of anxiety and other negative valence systems. Social interactions involve the willingness to spend time with a conspecific (Haller and Bakos, 2002; Lapiz-Bluhm et al., 2008; Reddy et al., 2024). We used similar procedures that were successful in assessing post-stroke depressive-like behaviors middle-aged female rats (Panta et al., 2019) and aligned with the RDoC domain for social processes (National Institute of Mental Health, 2019) and with our past work (Conrad et al., 2024).

The social exploration test involved determining whether the test rat preferred to spend time with a female novel conspecific (R1) compared to an inanimate object (O) as an index of sociability, then comparing the test rat preference for a familiar female conspecific (R1) to an unfamiliar female conspecific (R2) as an index of social novelty. The conspecifics R1 and R2 were OVX and similarly aged as the test rats and were counterbalanced between cage mates. The social interaction apparatus had three identical clear plastic chambers (32 liters), linearly connected by 2 PVC tubes (9 cm length, 11 cm diameter) to provide access to each of the chambers. Each chamber had a plastic lid to prevent escape and holes punctured throughout to permit air circulation. The sample objects and rat conspecifics were restricted to smaller boxes (64-ounce Glad food box, The Clorox Co.) with the lids punctured with holes for air circulation. The chambers, PVC pipes and boxes were cleaned before each trial with French Lavender scented, Method All-Purpose cleaner.

To begin, the test rat was acclimated and restricted to each of the three individual chambers for 10 min for a total of 30 min. Next, the test rat was allowed to explore the entire three chamber apparatus with unrestricted access through the inter-connecting PVC pipes for 10 min. For the first social interaction test, which assessed sociability, one end of the three-chamber apparatus contained a small box with a novel female conspecific (i.e., R1). The rats were able to see, hear, and smell each other, but without physical interactions. The other end of the three-chamber apparatus contained a similar box with an inanimate plastic object. The test rat was placed in the center chamber and allowed to explore all three chambers for 10 min. The sociability-index was computed as a percentage using the following for the test rat:

Time spent in the chamber with R1 ÷ Time spent in both side chambers (sum of time with R1 and O)

Following the completion of the sociability assessment, the next social interaction test measured an index of social novelty. R1 was placed in a new box and returned to the same location as before, becoming the “familiar” rat. The object was removed from the box and replaced with a novel female conspecific (R2). The test rat was placed in the center chamber and allowed to explore all three chambers for 10 min. The Social Novelty-index was computed as a percentage using the following for the test rat:

Time spent in the chamber with R1 ÷ Time spent in both side chambers (sum of time with R1 and R2)

A second analysis was performed in which the time spent with the test rat directing its attention toward the object, R1, and R2 was quantified and termed “interaction” behaviors. This quantification provided a more detailed analysis than simply assessing time in the overall chamber, as the test rat could be grooming or performing other non-social behaviors rather than engaging with the boxes containing the object/rats. An “interaction” was scored when the test rat pointed its head within 1 cm of the box containing R1, R2 or object and demonstrated active engagement (i.e., sniffing, whisker twitching). The test rat may also climb on top of the boxes, which was scored as an interaction if the test rat's head was directed toward the box and interaction behaviors were observed.

In contrast to the initial analysis of social interaction where time spent in the outer containers was scored for the full 10 min, the interaction behaviors were scored in two, 5-min bins. This binned analysis was performed to enhance the likelihood of detecting group differences in case rats quickly habituated to the environment.

### RDoC domain: negative valence behaviors (construct = potential harm or novelty-induced anxiety)

#### Defensive marble bury task

The MBT is thought to measure anxiety or neophobia by tapping into rodents' innate response to threat within their burrows/home-cages (De Boer and Koolhaas, 2003). Earlier work used a shock probe (Treit et al., 1981), but modifications now include using marbles in a variety of derivations (Ho et al., 2002; Pandey et al., 2009; Dagyte et al., 2011; Angoa-Peéez et al., 2013; Ku et al., 2016), with predictive validity to anxiolytic agents (Dulawa and Hen, 2005; Langer et al., 2020). Active (i.e., burying) and passive (i.e., freezing or immobility) coping behaviors were used to evaluate responses associated with anxiety-like behavior (de Brouwer et al., 2019).

Eight standard rodent housing cages were individually placed inside sound-attenuating cabinets that were purchased (Coulbourn, E10–23, 78.7 cm W × 53.3 cm D × 50.8 cm H) or custom-made (Melamine: 63.5 cm W × 61.0 cm D × 71.1 cm H) to allow the simultaneous testing of multiple rats. Each rodent cage was filled with regular Sani-Chip bedding to be at least 5 cm deep. The bedding was packed down to make it firm and evenly distributed. Marbles were placed on top of the bedding and distributed in a consistent pattern of three rows of four marbles for a total of 12 marbles clustered near one side of the rodent cage.

Rats were tested in squads of 7 to 8 rats. Each rat was placed in one of the prepared Sani-Chip bedding cages without marbles and allowed to explore for 10 min. Afterwards, the rats were removed, the bedding flattened, and marbles were distributed on top as described ensuring that about half the test cage was without marbles. The rats were returned to the section of the test cage furthest from the marbles and allowed to explore for 15 min while being recorded.

Behaviors quantified include the number of marbles buried by 2/3 and the time spent: (1) investigating marbles, (2) grooming, and (3) being immobile. Marble investigation was defined as the rat facing and/or interacting with a marble within 2 cm, but not actively burying it, and increased marble investigation was considered a lower anxiety-like profile. Marble burying, on the other hand, was considered an active anxiety-like behavior and was defined as actively moving the bedding in the direction of the marbles with either forepaws or hindlimbs. Grooming was considered a passive anxiety-like behavior and was defined as licking the body or actively moving forepaws on the face/body. Immobility was also considered a passive anxiety-like behavior and was defined as the absence of active movement, grooming, or interaction with the environment. Only the first 10 min of the test were analyzed, with the last 5 min excluded because behavior was indistinct between groups at that timepoint, likely due to acclimation to the environment.

#### Novelty suppressed feeding

NSF was utilized to assess anxiety profiles by measuring rats' willingness to consume food in an unknown and potentially threatening environment (Mitchell, 1976; Bodnoff et al., 1988; David et al., 2009; Snyder et al., 2011; Bocarsly et al., 2015; Blasco-Serra et al., 2017), consistent with our past work (Conrad et al., 2024). The NSF arena consisted of a black square field (96.5 cm × 96.5 cm) with high walls (38.1 cm) to prevent escape. The arena was in a novel room, brightly lit (170–180 lux), with most of the walls covered with curtains to reduce spatial cues. The arena was wiped down with Lime/Sea Salt scented Method All-Purpose cleaner. A pile of rat chow was in the center of the arena (4–5 pieces), which was discarded and replaced with fresh chow for each rat.

To begin, rats were food restricted overnight and still had *ad libitum* access to drinking water. The next day, a rat was placed in one corner of the arena and given 8 min to approach the food and to take the first bite. Latency to approach the food and to take the first bite were used as a measure of anxiety, with higher latencies indicating elevated anxiety. If the rat did not approach the food after 8 min, testing was terminated, and the rat was given a score of 480 sec. When completed, the rats were returned to the animal colony and housed individually for 10-min with pre-weighed chow available. The amount of chow consumed during the home-cage feeding was measured to assess motivation to eat when located in a familiar environment to control for the novel environment measures. Littermates were re-united after the end of the home-cage eating assessment and food was returned to *ad libitum* access.

### Composite scores

To compare disparate behaviors across behaviors, z-scores were computed and then combined. A z-score was calculated as follows:

Individual Score – Average Score for both treatments (VEH and E2 combined) ÷ The Standard Deviation for both treatments (VEH and E2 combined)

High values represented worse negative valence, anhedonia, disrupted social affiliation, and high anxiety. In some cases, the z-scores were multiplied by −1 to align across scores and this included scores for the SP index, SST grooming duration, social interaction (Sociability Index and time with R1 during sociability, Social Novelty Index and time with R2 during social novelty), and time interacting with marbles on the MBT. Scores that remained as calculated were immobility on the NSF, latency to approach food on NSF, and immobility and marbles buried on the MBT. A composite depressive score was created by adding the z-scores from FST (immobility), SST (time to initiate grooming), and time spent with R1 in the first 5 min of sociability and then dividing by 3. A separate composite z-score was created for anxiety because anxiety is not a symptom of depression but is often comorbidly expressed with it. The composite anxiety z-score was calculated using the sum of the z-scores for the NSF latency and time spent investigating marbles divided by two.

### Quantification and statistical analysis

Quantification of the FST, SST, Social Tests, and MBT were performed offline by hand. A minimum of two observers blinded to the experimental conditions scored each type of behavior. A scorer had to achieve at least a 95% intra-rater and inter-rater reliability to be used for quantification purposes. Individuals failing to meet the 95% reliability metrics were retrained until two scorers achieved at least 95% reliability.

The statistical software package SPSS (Version 28) was used to analyze the data. Levene's test for homogeneity of variance was used to ensure that the statistical analysis did not violate the ANOVA assumption of homogeneity/normality between groups, and then *t*-tests were performed as appropriate when comparing one dependent measure or a mixed factors ANOVA when several dependent measures were assessed. Effect sizes are reported with Cohen's d for two mean comparisons (Cohen, 2013) or with partial eta-squared following ANOVA analyses (Keppel, 1991; Lakens, 2013). In cases that ANOVA assumptions were violated, nonparametric Kolmogorov-Smirnov-tests were used. One rat died following surgery and so the final group sizes were as follows: VEH (*n* = 15) and E2 (*n* = 16) with two additional OVX rats used for the social testing. Data were represented as means (*M)* ± S.E.M. Statistical significance was defined when *p*-values were equal to or less than 0.05.

To compare across behaviors and different metrics, z-scores were computed using the following equation:

Z = (observed value – mean of the same)/standard deviation of the sample

In some cases, the z-score was multiplied by −1 so that high values would be consistent across measures. As an example, the z-score for the SP index was multiplied by −1 so that the highest values represented anhedonia and consistent with highest values from immobility in the FST. In cases where multiple measures were used, such as the MBT, the composite z-score is the sum of all z-scores, divided by the number of measures.

## Results

### RDoC domain: negative valence behaviors (construct = acute threat): FST

**Figure 2:**
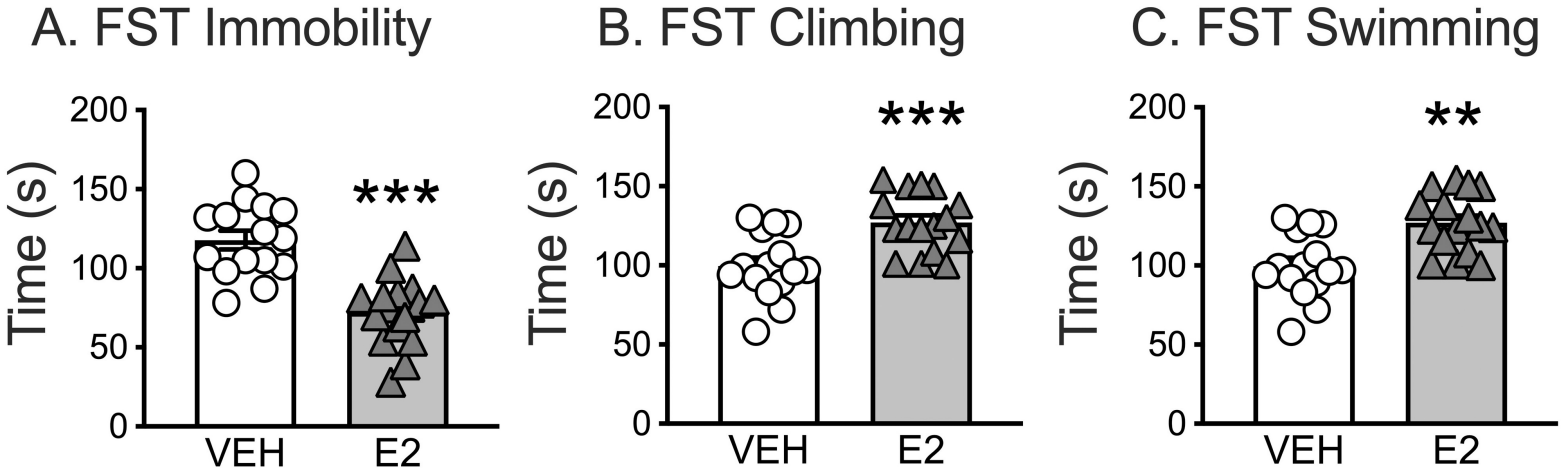
Forced swim test (FST) as a metric for negative valence for acute threat. E2 treatment **(A)** reduced the time spent immobile, **(B)** increased the time spent climbing, and **(C)** increased the time spent swimming. ** *p* < 0.01, *** *p* < 0.001. VEH *n* = 15, E2 *n* = 16.

E2 treatment significantly affected three key behaviors assessed during FST: immobility, climbing, and swimming. *T*-test comparisons revealed that E2 treated rats spent significantly less time immobile (72.4 ± 5.4 s) compared to VEH rats [117.8 ± 5.9 s, *t*(29) = 5.685, *p* < 0.001, *d* = 2.043]. E2 treated rats also spent more time climbing [E2 = 127.0 ± 4.7 s, VEH = 99.5 ± 5.3 s, *t*(29) = −3.891, *p* < 0.001, *d* = −1.399] and actively swimming [E2 = 17.6 ± 1.9 s, VEH = 10.4 ± 1.7 s, *t*(29) = −2.783, *p* = 0.009, *d* = −1.00] compared to VEH rats (Figure 2).

### RDoC domain: positive valence behaviors (construct = anhedonia): SP and SST

**Figure 3:**
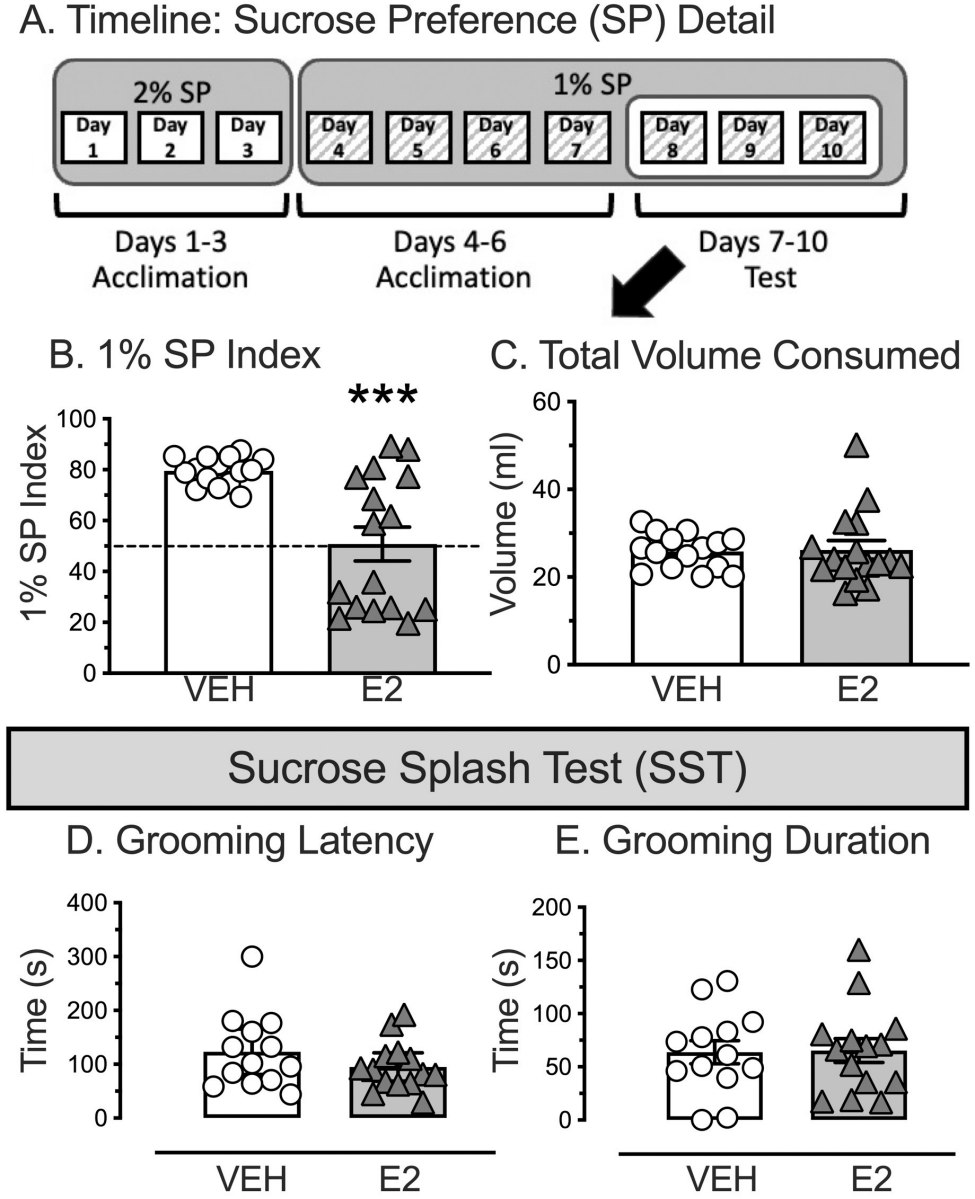
Sucrose preference (SP) timeline and sucrose splash test (SST) as a metric for positive valence for anhedonia. **(A)** Rats were exposed to a ten-day SP timeline that does not require drink or food deprivation. Rats were single housed and exposed to two drink bottles, 2% sucrose and drinking water for 3 days (denoted by white boxes) which was changed on the fourth day with 1% sucrose and drinking water for 7 days (denoted by gray hatched boxes). Testing was performed on the last 3 days of the 1% sucrose exposure. Bottle locations were counterbalanced, and locations swapped daily. **(B)** E2-treated rats drank significantly less 1% sucrose over the last 3 days of 1% SP testing compared to VEH-treated rats. **(C)** Total volume consumed during the last 3 days of 1% SP testing was similar between VEH and E2-treated rats. **(D)** After being splashed with 10% sucrose on the rats' backside, E2 treatment did not alter the latency to start grooming, nor **(E)** the total duration of grooming. *** *p* < 0.001. For SP, VEH *n* = 15, E2 *n* = 16. For the Splash Test, inadvertent disruptions reduced subject number to VEH *n* = 13, E2 *n* = 14.

E2 reduced the amount of sucrose consumed during the 3 days of final 1% SP testing without altering the total amount of liquid consumed (Figure 3A for Timeline). The SP index was analyzed by a non-parametric, independent samples Kolmogorov-Smirnov test due to unequal variances across groups, which violated the ANOVA assumption. E2 treatment reduced the consumption of 1% SP during the final 3 days of testing [D(31) = 1.913, *p* = 0.001, Figure 3B]. E2 treated rats had a significantly lower 1% SP index (44.4 ± 9.0) compared to VEH treated rats (62.1 ± 3.4), with a biphasic distribution that was not observed with VEH. Moreover, E2 treated rats drank similar amounts of sucrose (14.8 ± 3.0 ml/day) compared to water [11.4 ± 1.3 ml/day, paired *t-*test(15) = 0.830, *p* = 0.419, *d* = 0.208], while VEH rats consumed significantly more sucrose (20.7 ± 1.1 ml/day) compared to water [5.1 ± 0.3 ml/day, paired *t-*test(14) = 12.307, *p* < 0.001, *d* = 3.178]. Importantly, the overall fluid consumption during the final test phase was similar between groups (VEH, 77.5 ± 3.1 ml: E2, 78.6 ± 6.4 ml, Figure 3C) to support that E2 treatment reduced drinking of 1% SP without altering the total amount of liquid drank.

E2 and VEH treated rats showed similar grooming behaviors during the SST. After being splashed on their backside with 10% sucrose, VEH and E2-treated rats displayed similar latencies to initiate grooming [*t*(25) = 1.252, *p* = 0.222, *d* = 0.482, VEH = 123.1 ± 19.3 s, E2 = 95.0 ± 12.2 s, Figure 3D] and total duration spent grooming [*t*(25) = −0.099, *p* = 0.922, *d* = −0.038, VEH = 63.7 ± 10.9 s, E2 = 65.3 ± 11.1 s), Figure 3E]. Note, four rats were excluded (two in each group), due to inadvertent disruptions during testing.

To determine whether the SP and SST test may be related, a Pearson correlation was performed for the 1% SP index and the latency to initiate grooming in the SST. The outcome failed to find a significant effect (r = 0.032, *n* = 27, *p* = 0.874). Consequently, the measures for the SP and SST appeared to assess different constructs.

### RDoC domain: systems for social processes (construct = disrupted affiliation): social exploration

**Figure 4:**
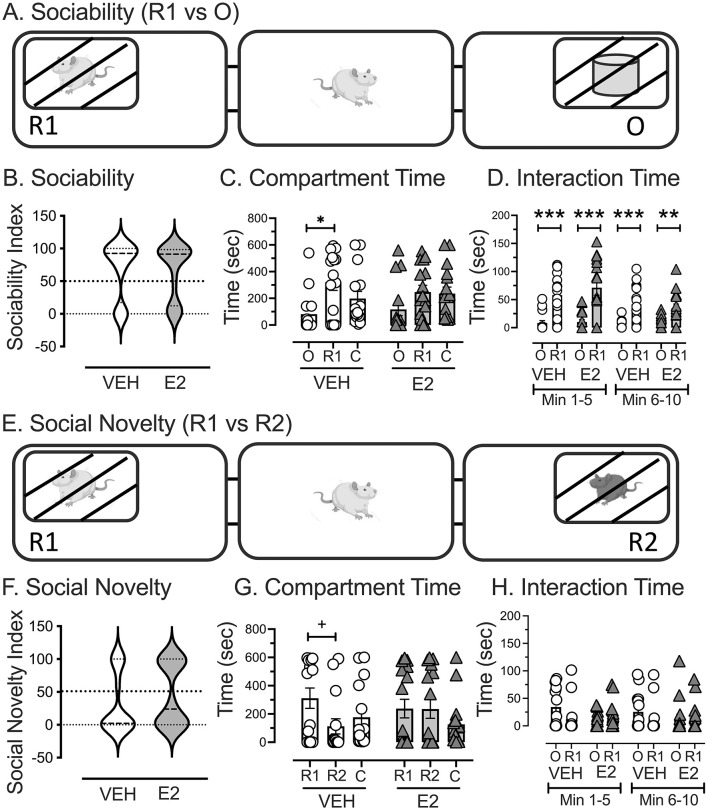
Assessment of E2 treatment on social interactions as a metric for social processes for disrupted affiliation. **(A)** The sociability test assessed whether the test rat spent time in the chamber with a novel conspecific female rat (R1) or an inanimate object (O). **(B)** The sociability index represented the time spent in the compartment with R1 compared to time in both side compartments and showed bimodal distributions that were statistically similar between VEH and E2. **(C)** VEH-treated rats spent more time in the chamber with R1 than with O, whereas E2-treated rats showed statistically similar times in both side chambers. **(D)** Quantifying the time that the test rat interacts with the box containing the R1 or the object showed that both E2 and VEH directed their attention more toward the R1 than the object in the first 5 min, which decreased in the second 5 min of assessment but still reached statistical significance. **(E)** The social novelty test assessed whether the test rat spent time in the chamber with R1 (familiar from the sociability trial) or a novel conspecific female rat (R2). **(F)** The social novelty index represented the time spent with R2 compared to time in both side compartments and showed bimodal distributions that were statistically similar between VEH and E2. **(G)** VEH-treated rats showed a tendency to spend more time in the chamber with R1 than with R2, but this did not reach statistical significance. E2-treated rats spent similar durations in both side chambers. **(H)** Quantifying the time that the test rat interacted with R1 and R2 showed no significant effect of E2 and VEH. C = center chamber, * *p* < 0.05, ** *p* < 0.01 *** *p* < 0.001, + *p* = 0.09 (with two-tailed, and <0.05 with one tailed paired *t*-test). VEH *n* = 15, E2 *n* = 16.

A sociability assessment was performed to determine whether an unfamiliar female rat or object was preferred (Figure 4A). Treatments were statistically similar for the sociability index when test rats could choose to spend time in a chamber containing a novel conspecific (R1) or an inanimate object, as revealed by the Kolmogorov-Smirnov test, which was used because the distributions were bimodally distributed [D(31) = 0.626, *p* = 0.828; Figure 4B]. Paired *t*-tests were performed for each treatment separately to determine whether test rats spent more time in the chamber with R1 or the object. VEH treated rats spent significantly more time in the chamber with R1 (320.1 ± 60.8 s) compared to the object [81.7 ± 39.7 s *t*(14) = 2.730, *p* = 0.016, *d* = 0.705], whereas the time spent in each side chamber did not reach significance for the E2 treated rats [R1 = 247.4 ± 49.7 s, O =116.9 ± 47.1 s, *t*(14) = 1.557, *p* = 0.140, *d* = 0.389, Figure 4C].

A separate analysis was performed to assess whether the test rat was interacting with the boxes containing the novel rat or object and quantified in the first 5 min separately from the second 5 min. A repeated measures ANOVA was performed for group (VEH, E2) across chambers (O, R1) and time range (Min 1–5, 6–10) for time spent interacting with the box containing the novel rat and object. A significant repeated effect of Time [*F*_(1, 29)_ = 16.009, *p* < 0.001, $\eta_{\text{p}}^{2}$ = 0.356], Chamber [*F*_(1, 29)_ = 38.197, *p* < 0.001, $\eta_{\text{p}}^{2}$ = 0.568], and Time by Chamber [*F*_(1, 29)_ = 11.924, *p* = 0.002, $\eta_{\text{p}}^{2}$ = 0.291] was found, with a three-way interaction approaching significance (*p* = 0.09), but failed to achieve statistical significance. There were no other significant effects. Both VEH and E2 treated rats interacted more with R1 than the object and both showed decreased interaction times in the second five-min bin compared to the first, while still interacting with R1 more than the object (Figure 4D).

A social novelty assessment was performed to determine whether the test rats preferred spending time in a chamber with a new unfamiliar conspecific female rat (R2) compared to a chamber with the previously exposed, female rat from the sociability assessment performed earlier (R1, Figure 4E). Treatments were statistically similar for the social novelty index, as revealed by a Kolmogorov-Smirnov test, which was used because the data were not normally distributed [D(31) = 0.812, *p* = 0.525, Figure 4F]. Paired *t*-tests were performed for each treatment separately to determine whether test rats spent more time in the chamber with R2 or R1. VEH treated rats tended to spend more time in the chamber with R1 (311.3 ± 71.7 s) than with R2 [112.2 ± 54.0 s, *t*(14) = 1.769, *p* = 0.099, one tailed *p* = 0.049, *d* = 0.456], which contrasted with E2 treated rats spending similar durations in both chambers [R1 = 238.1 ± 65.6 s, R2 = 235.4 ± 65.9 s, *t*(15) = 0.024, *p* = 0.981, Figure 4G]. To determine whether the test rats interacted with the boxes containing R1 and R2 differently, a repeated measures ANOVA was performed for Group (VEH, E2) across chambers (R1, R2) and time range (Min 1–5, 6–10) for time spent interacting with the box containing R1 and R2. No significant effects were found for any of the main effects and interactions (Figure 4H).

### RDoC domain: negative valence behaviors (construct = potential harm or novelty-induced anxiety): MBT and NSF

**Figure 5:**
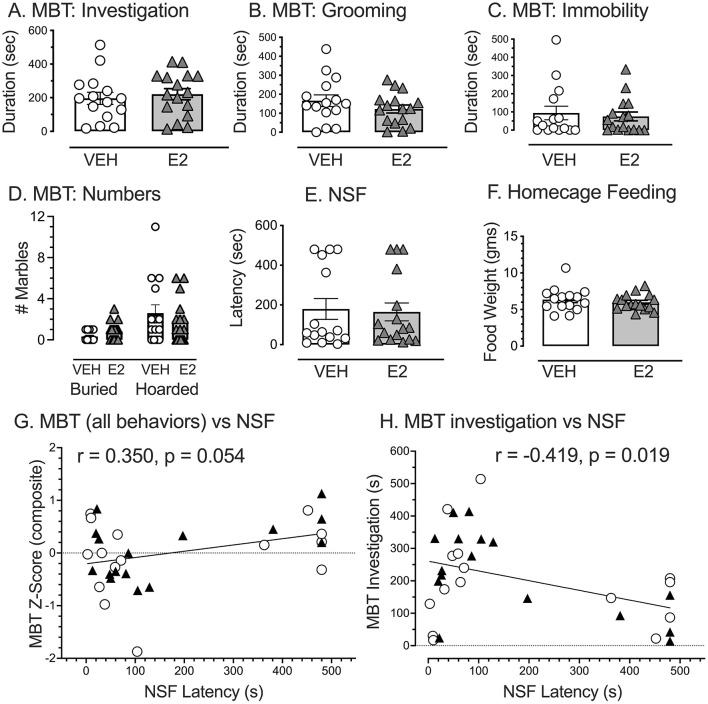
Marble bury task (MBT) and novelty suppressed feeding (NSF) as a metric for negative valence for potential threat. During the MB test, all five behaviors quantified failed to find significant effects of E2 on: **(A)** time spent investigating the marbles, **(B)** time spent grooming, **(C)** duration of immobility, **(D)** number of marbles buried by 2/3, and the number of marbles hoarded. For the NSF test, E2 had no effect on: **(E)** the time it took to approach food in the arena center and **(F)** amount of food consumed in the home cage. Notably, many rats never approached the food to create a ceiling effect. **(G)** A composite z-score for all the MBT behaviors (adjusted so that high z-scores indicated greater anxiety) marginally correlated with latency to approach food in the NSF (*p* = 0.054). **(H)** A negative correlation was significant when comparing the time spent investigating marbles on the MBT with time to approach food in the NSF (*p* = 0.019). Rats that showed high marble investigation tended to approach food quicker in the NSF. E2 and VEH treated rats were distributed throughout the correlation and did not fall within a particular region. White circles = VEH, Black triangles = E2. VEH *n* = 15, E2 *n* = 16.

During the MBT, E2, and VEH treated rats performed similarly in all variables assessed (Figures 5A–D). Student's *t*-tests revealed no significant difference between groups for the time spent investigating marbles [*t*(29) = −0.507, *p* = 0.616, *d* = −0.182, VEH, 196.1 ± 36.3 s: E2, 221.0 ± 33.1 s], time spent grooming [*t*(29) = 1.179, *p* = 0.248, *d* = 0.424, VEH, 165.9 ± 30.8 s: E2, 122.3 ± 21.2 s], time spent being immobile [*t*(29) = 0.467, *p* = 0.644, *d* = −0.168, VEH, 58.5 ± 25.8 s: E2, 75.0 ± 24.3 s], the number of marbles covered by two-thirds with the bedding [*t*(29) = −1.808, *p* = 0.081, *d* = −0.650, VEH = 0.33 ± 0.13, E2 = 0.81 ± 0.23], and the number of marbles hoarded [*t*(29) = 0.826, *p* = 0.416, *d* = 0.297, VEH = 3.1 ± 0.8, E2 = 1.8 ± 0.5].

For the NSF task, E2 treatment showed no effect on latency to approach food in the center of an arena (Figures 5E, F). A student's *t*-test revealed no significant difference between groups in the time it took to approach food in the center of the arena [*t*(29) = 0.214, *p* = 0.832, *d* = 0.077, VEH, 179.5 ± 52.2 s: E2, 164.8 ± 45.2 s, Figure 5E]. The distributions were bimodal in part, because many rats never approached the food to create a ceiling effect. Home cage feeding totals were corrected by body weight (mg consumed/g body weight) as groups weighed significantly different amounts at this point in the experiment. Groups showed similar home cage feeding behavior, consuming similar amounts of food per body weight during this duration [*t*(29) = 0.638, *p* = 0.529, *d* = 0.229, VEH, 6.4 mg ± 0.4 g: E2, 6.0 mg ± 0.3 g, Figure 5F].

To determine whether the assessments on the MBT and NSF latency measures may be related, Pearson correlation was performed on the composite MBT z-scores with the latency to approach food in the NSF. The correlation was marginally significant (r = 0.350, *n* = 31, *p* = 0.054) with a tendency for higher composite z-scores for MBT to correspond with longer latencies of rats to approach food in the center of the NSF (Figure 5G). A second correlation was performed on the time spent investigating marbles with the latency to approach food in the NSF. The correlation was significant and negatively correlated (r = −0.419, *n* = 31, *p* = 0.019), indicating that the rats spending more time investigating marbles on the MBT were more likely to quickly approach food in the NSF (Figure 5H). The rats in the VEH or E2 treatments appeared to be similarly distributed. These findings suggest that time spent investigating marbles and time to approach food in the NSF may involve similar constructs. Consequently, the negative valence for novelty-induced anxiety was computed using z-scores from the time investigating marbles in the MBT (multiplied by −1) so that high z-scores reflected high negative valence) and the latency to approach the food in the NSF.

### Across test comparisons

**Figure 6:**
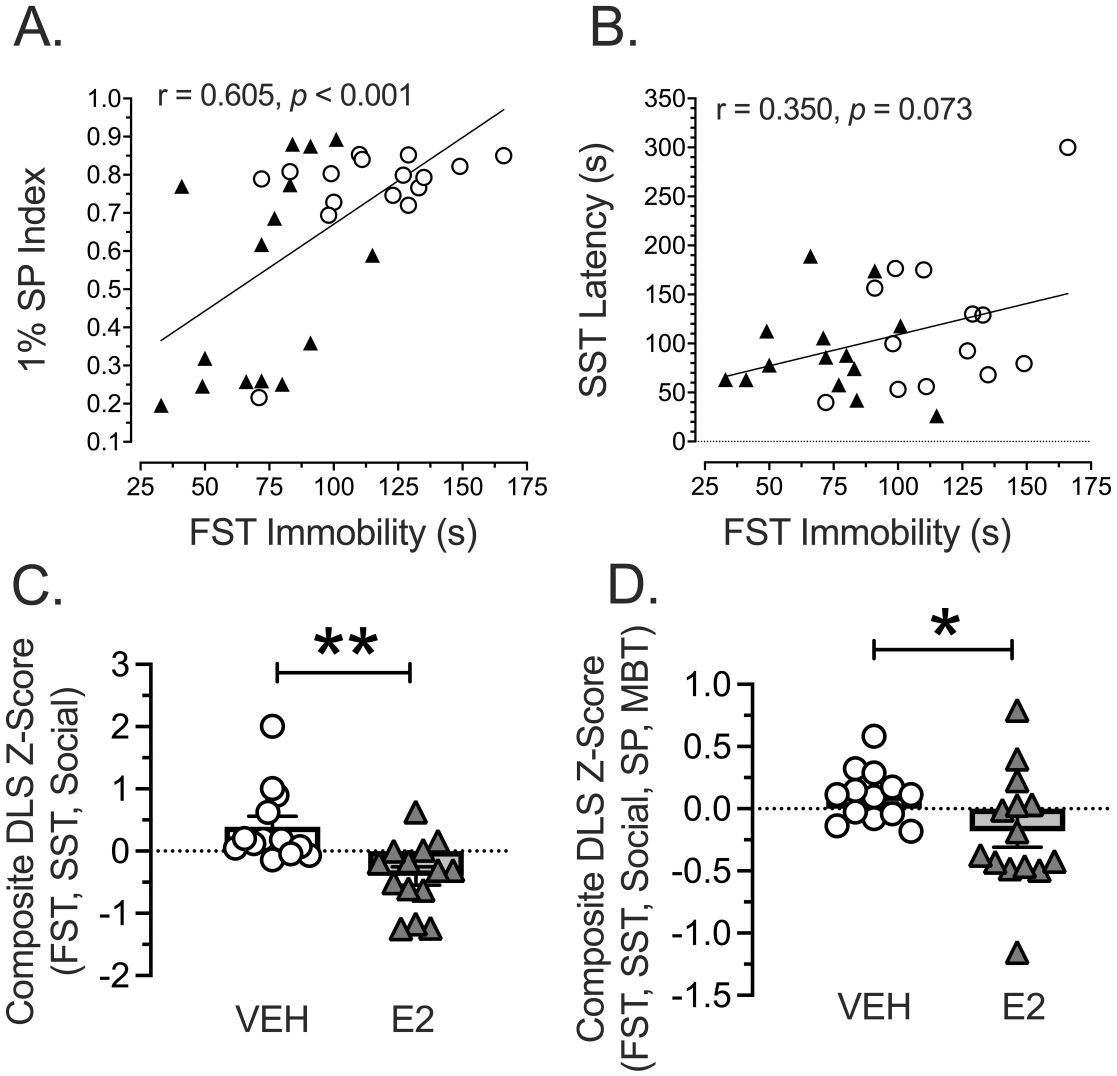
Comparison of task assessments. **(A)** Comparing the negative valence-acute threat assessment of immobility on the FST with the positive valence assessment using 1% SP Index revealed a significantly positive correlation (*p* = 0.002): Rats showing the highest immobility on the FST consumed the most sucrose, which was in the opposite direction as expected. E2 treated rats tended to cluster in the lower left to reflect reduced immobility with higher anhedonia associated with a low SP Index. VEH *n* = 15, E2 *n* = 16. **(B)** Comparing the negative valence-acute threat assessment of immobility on the FST with the positive valence assessment using the time to initiate grooming after a sucrose splash on the backside showed a near significant correlation (*p* = 0.072): Rats showing the highest immobility on FST took the longest to initiate grooming on the SST, which was in the predicted direction. VEH *n* = 13, E2 *n* = 14 (due to loss of rats from splash test malfunction). **(C)** A composite DLS z-score was computed for the FST immobility, SST to start grooming, and the Sociability test for interacting with the novel rat in the first 5 min was computed and adjusted so that higher z-scores reflected DLS. A *t-*test was significant (** *p* < 0.01): E2-treated rats showed a significantly lower composite DLS z-score compared to VEH-treated rats to indicate that E2 lowered DLS. VEH *n* = 13, E2 *n* = 14. **(D)** A composite DLS z-score was computed for all metrics using immobility (FST), 1% SP Index (SP), duration to initiate grooming (SST), time interacting (1^st^ 5 min Sociability), and duration investigating (MB) and adjusted so that higher z-scores reflected DLS. A *t*-test was significant (* *p* < 0.01): E2-treated rats showed a significantly lower composite DLS z-score compared to VEH-treated rats to indicate that E2 lowered DLS. VEH, White Circles; E2, Black Triangles. SP, sucrose preference; SST, sucrose splash test; FST, forced swim test; MBT, Marble Bury Task.

Pearson correlations were performed to determine whether performance on different assessments may be related. The negative valence of the acute threat measure of immobility in the FST was compared with the positive valence assessments, which were performed separately because the 1% SP and latency to initiate grooming with SST did not correlate. The correlation for FST immobility and 1% SP Index was significantly and positively correlated (r = 0.605, *n* = 31, *p* < 0.001). Unexpectedly, rats showing the highest immobility on the FST correlated with the highest amount of 1% sucrose consumed with VEH-treated rats falling near the upper right quarter of the graph and the E2-treated rats showing a bimodal distribution (Figure 6A). For the Pearson correlation comparing the FST immobility with the duration to initiate grooming on the SST, the effect approached significance (r = 0.350, *n* = 27, *p* = 0.073). Rats showing higher immobility on the FST also tended to take longer to initiate grooming after being splashed with sucrose, which followed the expected direction (Figure 6B). Consequently, a composite z-score was computed, based upon immobility on the FST and latency to start grooming on the SST, which were combined with the z-scores for the sociability test calculated from the time spent investigating the novel rat during the first 5 min. High z-scores indicated high negative valence and social withdrawal in a DLS metric. A *t-*test showed a significant effect [*t*(25) = 3.555, *p* = 0.0015, *d* = 1.369, Figure 6C]. E2-treated rats showed a significantly lower composite z-score compared to VEH-treated rats, indicating that E2 reduced the negative valence and anhedonic profile from the combined acute threat and hedonic assessments.

Since women show high comorbidity for depression and anxiety, another comparison was performed in the rodent model using the composite DLS z-score with the composite anxiety z-score. A Pearson correlation was not significant. However, the VEH-treated rats tended to show a different pattern than the E2-treated rats, and so a separate Pearson correlation was performed for each treatment. A Pearson correlation revealed a highly significant effect for VEH (r = −0.716, *n* = 13, *p* = 0.006) with no significant effect for E2. VEH-treated rats with the highest composite DLS z-scores showed the lowest composite anxiety profiles, which was in the unexpected direction. Finally, an over-arching composite z-score was computed to represent immobility (FST), 1% SP Index, latency to initiate grooming (SST), social interactions (1^st^ 5 min of Sociability) and duration investigating marbles. NSF was not included because of the ceiling effects observed and because the constructs assessed by this task and the MBT appeared to be related. Due to unequal distributions, A Kolmogorov-Smirnov test was performed and found to be significant [D(27) = 0.500, *p* = 0.0417, Figure 6D]. E2 lowered the DLS compared to VEH-treated rats to reflect low negative valence for acute threat and perceived threat (anxiety), as well as increased positive valence and sociability.

### Body weight

**Figure 7:**
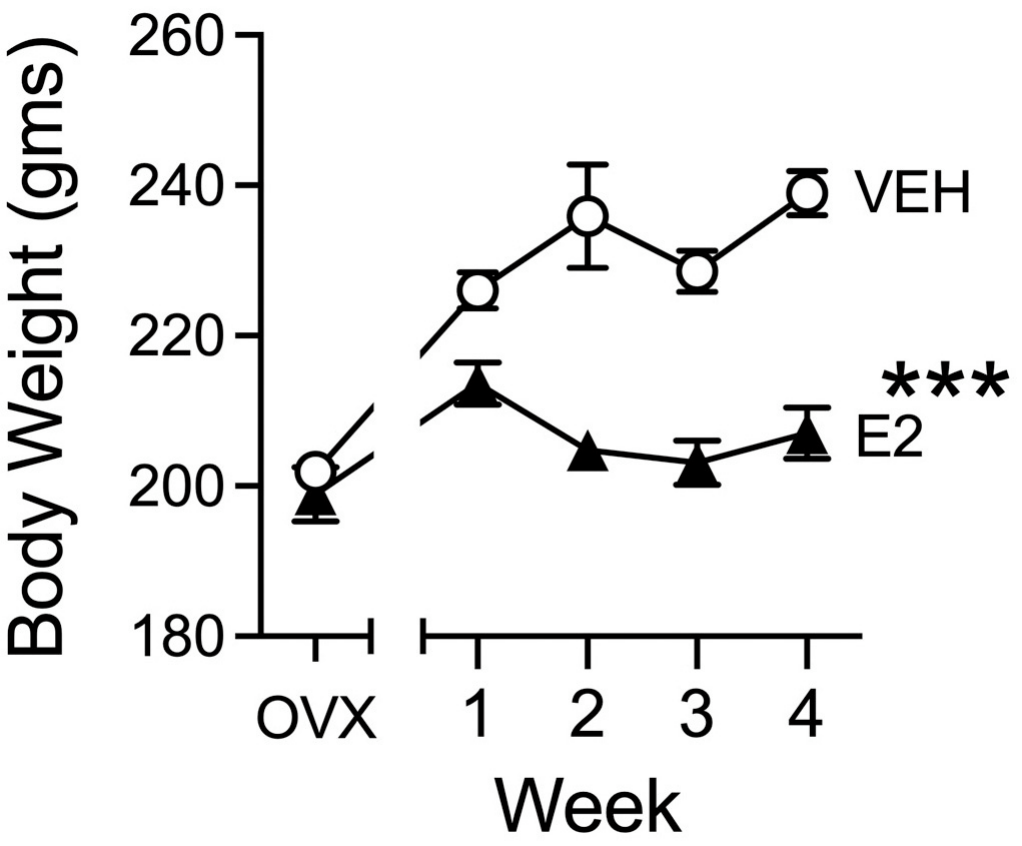
Body weight changes during treatment. VEH and E2 groups had similar in body weights at the time of OVX and before treatments began to indicate no *a-priori* differences. After several weeks, E2 or VEH treatments began and during the weekly weight measures, rats treated with E2 (3 μg, s.c., daily) lost weight during the initial 2 weeks of E2 and then stabilized. VEH-treated rats showed a pattern of gaining weight during the same duration. VEH *n* = 15, E2 *n* = 16. Note, error bars are plotted for all points but in some cases, may be smaller than the symbol size. ***p < 0.001.

E2 treatment reduced weight gain throughout the experiment (Figure 7). Body weights between the two groups before E2 treatment began were similar at the time of OVX surgery [*t*(29) = 0.682, *p* = 0.500, *d* =0.245, VEH, 201.9 ± 2.3 gms: E2, 198.9 ± 3.6 gms], to indicate that there were no differences prior to E2 treatment. Once E2 treatment began, E2 reduced body weight, when assessed by a repeated measures ANOVA across 4 weeks of E2 treatment, which revealed a significant main effect of group [*F*_(1, 29)_ = 35.574, *p* < 0.001, $\eta_{\text{p}}^{2}$ = 0.551] and a significant effect of group across weeks [*F*_(1, 29)_ = 28.757, *p* < 0.001, $\eta_{\text{p}}^{2}$ = 0.498] with no significant effect of week. Once E2 treatment began, body weights decreased by the 2^nd^ week of treatment and stayed low for the duration of the E2 treatment, whereas VEH-treated rats tended to gain weight during the same four-week duration.

## Discussion

The current study investigated whether E2 had anti-depressant qualities, based upon the RDoC metrics, during the early phase of middle-age when females are most susceptible to mood disorders (Schmidt and Rubinow, 2009; Soares, 2017). Since depression is complex and involves different symptoms (Gururajan et al., 2019; National Institute of Mental Health, 2019; Slattery and Cryan, 2014) and is unlikely to be captured in a single rodent model (Gencturk and Unal, 2024), we focused on three core valence features of depression: negative valence (acute threat), positive valence (anhedonia), and social assessment (disrupted affiliation). The behavioral tests used to explore these key features of DLS included: (1) FST for acute threat, (2) SP and SST for anhedonia, and (3) social exploration for disrupted affiliation. We additionally assessed the MBT and NSF to assess negative valence (i.e., potential threat or novelty-induced anxiety) as a metric for anxiety-like behaviors. We found that E2 treatment had seemingly opposing actions on valence states: E2 decreased FST immobility to suggest improved negative valence, but reduced SP to suggest anhedonia. Moreover, rats showing the least FST immobility expressed the lowest interest in sucrose. These findings may indicate that these processes have neurobiological underpinnings that are differentially influenced by E2, although we acknowledge that immobility in the FST can be differentially interpreted as an adaptive behavior, suggesting that these findings may be aligned. However, the FST task is still commonly utilized to screen antidepressant drugs due to its high predictive validity and reliability in showing reduced immobility following antidepressant administration (Petit-Demouliere et al., 2005). While we suggest caution when interpreting our FST findings, they are aligned with the expected outcomes for antidepressant efficacy (Brandwein et al., 2023). For the other behavioral tasks (SST, Sociability, MBT, and NSF), E2 had no significant effect or showed non-significant patterns in the predicted direction to suggest minimal improvements. Based upon the recommendation for preclinical work on depression (von Mücke-Heim et al., 2023), composite DLS z-scores were calculated being restrictive (from FST, SST, and social test) and inclusive (FST, SP, SST, MBT, and social) and both revealed that E2 significantly reduced the DLS metric in OVX middle-aged rats (Figures 6C, D).

von Mücke-Heim et al. (2023) recommended several criteria for assessing DLS in preclinical work. First, several measures should be factored into the DLS instead of relying upon one test, which we accomplished by creating a composite z-score from the FST, SST, and social interaction tests and more inclusive with five tests. The FST was used in the restrictive DLS because it aligned with the patterns observed with the SST and sociability measures. Second, the duration of the DLS should be at least 7 days for a murine model our study tested the same rats over several weeks. Third, the *p*-value and effect size should be at least *p* < 0.05 and Cohen's *d* ≥ 0.5, respectively. We easily met this threshold with OVX producing a DLS of *p* = 0.0015 and *d* = 1.369 (Figure 6C), which was consistent with OVX producing a depressive-like state in other reports (Gogos et al., 2018; Renczes et al., 2020). Fourth, we did not meet the criteria of four symptoms being demonstrated, finding opposite effects on the FST and SP, as well as non-significant patterns toward the predicted direction for SST and the MBT. Altogether, the findings support the interpretation that E2 improves the DLS observed in OVX middle-aged female rats, but with significant effects that can be described as moderate.

A potential reason why E2 effects were moderate, especially when each assessment is viewed in isolation (i.e., out of context from the other tasks) could be that E2 was applied to an “uncompromised” state. In other words, the effects of E2 could have been more robust when studied in a DLS model, such as that produced by chronic stress (Willner et al., 1992; D'Aquila et al., 1994; Gregus et al., 2005; Bondi et al., 2008; Duman et al., 2016; Seewoo et al., 2020) or chronic exposure to the stress steroid, corticosterone (Gregus et al., 2005; Ardayfio and Kim, 2006; Ding et al., 2018; Ngoupaye et al., 2018; Brymer et al., 2020; Nickle et al., 2020; Conrad et al., 2024). Supporting this interpretation is the previous finding that the removal of endogenous estrogens by OVX increased immobility on the FST in young (3 mos) and mature (7–8 mos) rats, but not in middle-aged female rats (Kiss et al., 2012). Consequently, E2 likely attenuated DLS in our study because we used a variety of assessments and created a composite DLS z-score to increase our ability to detect potential effects.

The FST and SP were implemented in the current study because of extensive prior work using these tasks and the ability to compare different behavioral assessments in the same subjects. The current findings are consistent with prior studies reporting that E2 has antidepressant properties on the FST (Rachman et al., 1998; Estrada-Camarena et al., 2003; Koss et al., 2012; Vega-Rivera et al., 2013, 2016), but differ from those that show E2 to have antidepressant effects on the SP (Romano-Torres and Fernandez-Guasti, 2010; Cheng et al., 2013; Gogos et al., 2018). While fewer reports include both the FST and SP tasks when assessing estrogen's actions in the same subjects, some findings are notable. In one study, OVX increased immobility on the FST and lowered the SP Index in mice, demonstrating that the loss of endogenous estrogen induces depressive-like behaviors on both tests (Zhang Z. C. C. et al., 2023). In another, E2 had antidepressant-like properties on the SP and FST following myocardial infarction (Najjar et al., 2018). However, another study reported mixed outcomes, similar to what we observed, with E2 having antidepressant qualities on the SP without affecting performance on the FST in young adult OVX rats (Gogos et al., 2018). In the Gogos et al. (2018) study, saccharin was used instead of sucrose for the SP test because E2 can alter food intake and metabolism (Butera, 2010; Gogos et al., 2010). As such, the SP test using sucrose in our study may have confounded our results, and also adds further support for the use of the FST in addition to the SP test when assessing estrogen's actions.

For the social exploration test, the results were mixed for sociability and social novelty. For sociability, both VEH and E2-treated OVX middle-aged rats showed a preference toward a conspecific over an object, which aligned with the literature showing that rodents typically explored a conspecific more than an inanimate object or an empty arena (File and Seth, 2003; Moy et al., 2004; Nadler et al., 2004; Lapiz-Bluhm et al., 2008; Ku et al., 2016; Panta et al., 2019; Hackenberg et al., 2021). Moreover, sociability interest wanes across the span of 10 min (Figure 4D), which is consistent with prior findings of decreased conspecific exploration with repeated exposure (Spiteri and Agmo, 2009). In contrast, our findings differed from the majority of the literature on social novelty by our rats failing to show a preference for a novel conspecific over a familiar one (Moy et al., 2004; Nadler et al., 2004; Boyer et al., 2019; Hackenberg et al., 2021). Nonetheless, our work aligned with a report finding that OVX rats preferred cage-mates over novel conspecifics (Garcia et al., 2017). These different findings across studies could reflect variations in sociability across rat strains (Ku et al., 2016; Beery and Shambaugh, 2021). Another intriguing possibility is that novelty-seeking behaviors change with age and peak in early to mid-adulthood (Markham and Juraska, 2007; Vetter-O'Hagen and Spear, 2012; Arias-Cavieres et al., 2017; Koebele et al., 2020; Canatelli-Mallat et al., 2022). This is supported by prior work showing that rodents failed to explore novel objects or novel conspecifics when test subjects were middle-age for females or males (Perkins et al., 2016; Koebele et al., 2020) and in pre-adolescent rats (20 day old, McKibben et al., 2014). However, social novelty was detected following puberty in rodents (Vetter-O'Hagen and Spear, 2012), with a decline observed by 9-months of age (Boyer et al., 2019). As novelty exploration may start to wane around the age of 9-months (Perkins et al., 2016), this suggests that social novelty is non-linear and may not be an ideal measure for older rodents.

The anxiety assessments were notable in that E2 failed to alter behavior in the MBT and NSF. Studies showed that E2 (3 μg or 5 μg) is commonly anxiolytic on the OF and EPM (Frye and Walf, 2004; Hiroi et al., 2016), although we previously found that 5 μg of E2 was ineffective at altering OF performance in OVX young adult female rats (McLaughlin et al., 2008). One possibility is that different cognitive processes may require unique levels of E2 exposure, as 3 μg dose of E2 was selected based upon past work showing spatial working memory improvements with this dose (Koebele and Bimonte-Nelson, 2015; Prakapenka et al., 2018). Another possibility is that behavioral testing order could have masked some outcomes because anxiety testing in our study was performed after SP and SST, compared to other studies that assessed anxiety first when using E2 (Frye and Walf, 2004). Although Hiroi et al. (2016) still found E2 effects on anxiety when measured after water maze testing, testing order could have contributed to these findings because the aversive nature of the task can elevate plasma stress hormone levels of corticosterone (Harrison et al., 2009). Regarding, the z-scores for time investigating marbles and the latency to approach food in the NSF, these scores were significantly and negatively correlated (Figure 5H), to suggest that these measures were assessing common underlying constructs in a DLS phenotype. Moreover, we observed a novel behavior, where the rats often played with and hoarded the marbles, representing atypical behaviors for aversive stimuli (Poling et al., 1981; Fucich and Morilak, 2018) and unreported except for mention from pilot work (Ku et al., 2016). Perhaps playing/hoarding behavior represents a unique, age-specific change in females that will require further empirical assessment. Alternatively, these findings may be interpreted as a time-dependent shift in avoidance-behaviors (i.e., neophobia) toward approach behaviors (i.e., neophilia), as the marble stimuli became less novel with prolonged exposure (Hughes, 2007). Future investigations could thus utilize an abbreviated testing period to prevent habituation to these stimuli or conduct binned analyses to determine changes in avoidance- and approach- behaviors across time. Additionally, we failed to find a significant relationship between the composite z-scores for DLS and anxiety. This may be anticipated because anxiety is highly comorbid with depression (Kalin, 2020), suggesting that the focus should solely be on the group expressing DLS (i.e., VEH-treated OVX rats). Indeed, VEH-treated rats with the highest composite z-scores for DLS showed the lowest anxiety profile, which was the opposite of what we expected and corroborates the interpretation that rats may need to be exposed to a stressor to observe anxiety profiles. Finally, prior findings that prolonged corticosterone exposure produced moderate effects on affective behaviors assessed by the EPM, OF, NSF, and SST in young adult female mice (Mekiri et al., 2017), as well as the current observation that our rats played with the marbles, suggests the possibility that these tasks were not as aversive as intended for our cohort of animals.

Several neurobiological mechanisms may underlie E2 antidepressant effects. Estrogen can modulate multiple neurotransmitter systems like serotonin and dopamine, which can subsequently affect mood regulation, motivation, and pleasure (Hwang et al., 2020). Estrogen also increases dendritic spine density and neurogenesis in the hippocampus, akin to the effects of standard antidepressant treatments (i.e., Selective Serotonin Reuptake Inhibitors, SSRI) that are used to mitigate the reduced hippocampal volume found in individuals with major depressive disorder (Wharton et al., 2012). In depressed premenopausal and menopausal women taking SSRIs, estrogen supplementation to downregulate postsynaptic serotonin receptors is necessary to achieve similar levels of symptom relief in comparison to tricyclic antidepressants without estrogen replacement (Parry, 2010). Moreover, exogenous estrogen administration may slow or even prevent hippocampal changes that occur with the depletion of estrogen during menopause in humans (Wharton et al., 2012). These effects of estrogen may be further explained by its ability to increase neurotrophins (e.g., brain-derived neurotrophic factor, BNDF), which promote neuroplasticity and neuroprotection (Hwang et al., 2020). Together, these findings demonstrate several mechanisms by which estrogen may induce antidepressant-like effects, but further investigations are needed to identify the specific mechanism underlying these changes.

While beyond the scope of the current investigation, future studies could examine the specific neurotransmitter systems affected by the RDoC in question to improve understanding of the underlying mechanisms and outcomes in clinical settings. Prior research has found that women tend to respond better to SSRIs while men respond better to tricyclic antidepressants (TCAs). Consequently, future studies could examine how underlying biological differences in the serotonergic system and in fluctuating estrogen levels may modulate such sex-specific differences in treatment outcomes (Young et al., 2009; Carvalho Silva et al., 2023).

Some limitations of the current study need to be addressed. First, we used an OVX model, which may limit translation to humans because few women undergo complete hormonal depletion through similar procedures like bilateral oophorectomy. However, we believe that using an OVX blank state model has the benefit of assessing estrogen actions without endogenous estrogens confounding interpretation (Koebele and Bimonte-Nelson, 2016). Second, behavior on the FST may reflect altered coping strategies instead of helplessness (Molendijk and de Kloet, 2015; Armario, 2021). Nonetheless, the FST results aligned with the patterns we observed with the SST and sociability to provide corroborative data in our study and also has antidepressant treatment predictive validity (Reddy et al., 2024). Third, a single low E2 dose (3 μg daily injections) was used instead of a range of doses. As this was a first pass to assess E2 actions on DLS, the 3 μg dose (as opposed to 5 μg/rodent, 10 μg/rodent, or 0.5 mg/kg; see McLaughlin et al., 2008; Koebele and Bimonte-Nelson, 2015; Li et al., 2023) was selected based upon prior work's success (Koebele and Bimonte-Nelson, 2015; Prakapenka et al., 2018), with dose ranges to be targeted in the future. Fourth, rats were injected daily with E2, instead of cyclically (McLaughlin et al., 2008), which raises a concern that behavioral testing completed near the end of the study may have reflected higher cumulative E2 exposure. However, the evidence shows that daily administration of oral estrogens increased in the first 5 to 10 days and then stabilized thereafter, with a recommendation of a 24-h dosing interval (Stanczyk et al., 2013), to suggest that E2 did not accumulate. Fifth, as estrogen metabolism can change with age (Kwekel et al., 2010; Stanczyk et al., 2013), this may be an important factor in the assessment of E2 actions near the beginning of middle-age and in the differences across past studies. Sixth, some distributions were bimodally spread (i.e., SP, sociability), with reasons that are currently unknown.

The current study demonstrated that middle-aged, OVX female rats treated with a low dose of E2 that successfully improved cognitive performance in past studies (Koebele and Bimonte-Nelson, 2015; Prakapenka et al., 2018), also produced an anti-DLS phenotype without influencing anxiety-like behaviors. We created a broad composite z-score for DLS and a targeted z-score using metrics that showed similar patterns: immobility on the FST to represent negative valence (acute threat), time to initiate grooming on the SST to represent positive valence (anhedonia), and time spent with attention directed toward a novel conspecific to represent social assessment (disrupted affiliation). Both the broad and targeted composite DLS z-scores revealed that E2 had positive valence-boosting properties in early, middle-aged, OVX female rats. For E2′s effect on anxiety profiles, no effect was found. Given that E2′s actions overlap with those involved in stress (Albert and Newhouse, 2019), future studies should investigate the effects of E2 on DLS using rodents compromised by chronic stress. In summary, early, middle-aged OVX females show a DLS that can be significantly improved with E2 when provided at a constant, low dose.

## Data Availability

The raw data supporting the conclusions of this article will be made available by the authors, without undue reservation.
